# Prognostic Factors and Implantable Cardioverter-Defibrillator Outcomes in Transthyretin Cardiac Amyloidosis: A Comprehensive Retrospective Study

**DOI:** 10.31083/RCM39760

**Published:** 2026-01-20

**Authors:** Mohammed Alaa Raslan, Hussein Abdul Nabi, Nour B. Odeh, Mayar H. Alatout, Omar Baqal, Mohammed Tiseer Abbas, Hicham Z. El Masry, Dan Sorajja

**Affiliations:** ^1^Department of Cardiovascular Medicine, Mayo Clinic Arizona, Phoenix, AZ 85054, USA; ^2^Department of Cardiovascular and Thoracic Surgery, Mayo Clinic Arizona, Phoenix, AZ 85054, USA; ^3^Department of General Surgery, Mayo Clinic Arizona, Phoenix, AZ 85054, USA

**Keywords:** amyloid 1, transthyretin cardiac amyloidosis 2, cardiomyopathy 3, implantable cardioverter defibrillators 4, defibrillators 5, mortality 6, cardiac arrhythmia 7, sudden cardiac death 8

## Abstract

**Background:**

Transthyretin (TTR) cardiac amyloidosis is a progressive cardiomyopathy with high mortality; however, the role of implantable cardioverter-defibrillators (ICDs) in this population remains unclear.

**Methods:**

This retrospective cohort study included patients with confirmed TTR cardiac amyloidosis, with or without ICDs, from January 1, 2001, to December 31, 2024, across all three Mayo Clinic sites (Arizona, Florida, and Minnesota). Diagnosis was confirmed by endomyocardial biopsy or abnormal technetium pyrophosphate (PYP) scintigraphy. A 1:4 propensity score-matched cohort of non-ischemic cardiomyopathy (NICM) patients with ICDs served as a control group. The primary outcome was all-cause mortality, comparing transthyretin cardiac amyloidosis (TTR-CA) patients by ICD status and against matched NICM patients. Secondary analyses evaluated predictors of mortality, including the use of tafamidis and the indication for ICD (primary vs. secondary prevention). Kaplan–Meier and Cox regression analyses were used to assess predictors of survival and mortality.

**Results:**

A total of 463 patients with confirmed TTR cardiac amyloidosis were included. The median follow-up duration was 7.4 years (interquartile range (IQR): 5.3–9.2 years) for the non-ICD group and 6.8 years (IQR: 4.5–9.0 years) for the ICD group. The median age was 74.5 years (IQR: 68.0–80.0 years), and 92.9% of patients were male. Among them, 206 (44.5%) received ICDs and 257 (55.5%) did not. ICD recipients were younger (71.0 vs. 77.0 years; *p* = 0.001) and had higher rates of hypertension (62.6% vs. 45.6%; *p* = 0.001), chronic kidney disease (CKD) (62.6% vs. 44.4%; *p* = 0.001), and diabetes (30.1% vs. 21.8%; *p* = 0.043). Median left ventricular ejection fraction was lower in the ICD groups (43% vs. 54%; *p* = 0.007), and N-terminal pro-B-type natriuretic peptide (NT-proBNP) levels were higher in the ICD group (2259.0 pg/mL vs. 1503.0 pg/mL; *p* = 0.007). Among ICD recipients, 157 (76.2%) received the device for primary prevention, while 48 (23.3%) received the ICD for secondary prevention. Appropriate shocks were delivered in 22 patients (10.6%), primarily for ventricular tachycardia (n = 18) and ventricular fibrillation (n = 4). Inappropriate shocks occurred in six patients (3.0%), and 12 patients (5.8%) experienced device-related complications. Over 10 years of follow-up, ICD implantation did not confer a survival benefit for patients with TTR-CA compared to those without an ICD (*p* = 0.74). In contrast, a 1:4 propensity-matched NICM cohort with ICDs, which had a median follow-up of 7.1 years (IQR: 4.6–8.8 years), showed significantly improved survival than TTR-CA patients with ICDs (*p* = 0.034). Among the TTR-CA patients with ICDs, neither the use of tafamidis (*p* = 0.10) nor the ICD indication (primary vs. secondary prevention; *p* = 0.85) influenced mortality. In the Cox regression analysis, predictors of mortality in TTR-CA patients included older age (hazard ratio (HR) 1.048; *p* = 0.001), CKD (HR 1.637; *p* = 0.029), troponin T >50 ng/L (HR 1.594; *p* = 0.031), NT-proBNP >3000 pg/mL (HR 1.514; *p* = 0.050), and ejection fraction <40% (HR 1.935; *p* = 0.003). ICD implantation was not associated with improved survival (HR 0.932; *p* = 0.763).

**Conclusions:**

In conclusion, our data suggest that ICD therapy may not provide a significant overall survival benefit in older TTR-CA patients with impaired pump function; thus, prospective studies are warranted before any changes to clinical practice are considered. Key predictors of mortality included reduced ejection fraction and elevated cardiac biomarkers. Additional prospective studies are needed to clarify the role of ICDs in treatment strategies for patients with TTR-CA.

## 1. Introduction 

Transthyretin (TTR) cardiac amyloidosis is a progressive, infiltrative cardiomyopathy characterized by the extracellular deposition of misfolded TTR protein in the myocardium. It can be caused by either wild-type TTR (ATTRwt) or variant TTR (ATTRv) due to genetic mutations. TTR cardiac amyloidosis has been increasingly recognized as a significant cause of cardiomyopathy with progressive heart failure and increased mortality in the elderly [1].

The diagnostic gold standard for cardiac amyloidosis remains endomyocardial biopsy with proteomic typing by mass spectrometry. However, since 2016, non-invasive diagnosis of TTR cardiac amyloidosis has become possible in patients without evidence of a monoclonal protein, using technetium-99m pyrophosphate (PYP) scintigraphy with single-photon emission computed tomography (SPECT) imaging. In the presence of a monoclonal protein, a tissue diagnosis with mass spectrometry remains essential for confirming amyloid subtype. While cardiac magnetic resonance (CMR) imaging cannot differentiate between amyloid subtypes, it plays a critical role in detecting infiltrative cardiomyopathy and supports the diagnostic process [2].

Mortality rates in TTR cardiac amyloidosis vary based on subtype and disease stage. In the Transthyretin Amyloidosis Cardiac Study (TRACS), median survival from diagnosis was 25.6 months for patients with the V122I mutation, compared to 43.0 months for those with wild-type TTR. Mortality rates can be significantly higher in advanced stages or in the presence of certain risk factors [3]. Current indications for implantable cardioverter defibrillator (ICD) implantation in patients with TTR cardiac amyloidosis are not well-established and remain a topic of ongoing debate [4, 5]. The decision to proceed with ICD placement is typically individualized, considering multiple factors including the patient’s indication for ICD therapy (primary or secondary prevention), anticipated life expectancy, burden of comorbid conditions, and personal values or preferences regarding quality of life and invasive therapies [4].

The literature has identified several predictors of early death in TTR cardiac amyloidosis. These factors outperformed the New York Heart Association (NYHA) functional class in predicting 18-month mortality [6]. A systematic review and meta-analysis in 2020 identified additional prognostic factors, including right ventricular dysfunction, low voltage on electrocardiogram (ECG), and pericardial effusion. The authors proposed a risk score incorporating these factors to predict short-term mortality in TTR cardiac amyloidosis but still concluded that the frequency of appropriate ICD treatment in cardiac amyloidosis is low and is not predicted by non-sustained ventricular tachycardia (VT) [7].

Despite the increasing recognition of cardiac amyloidosis and its associated high mortality risk, there remains a significant gap in the literature regarding the utility of ICD implantation to reduce mortality, as existing evidence remains inconclusive due to small and heterogeneous study populations, lack of randomized trials, short follow-up, and inconsistent use of guideline-directed indications. Existing research has failed to demonstrate a clear mortality benefit from ICD use in cardiac amyloidosis patients [7]. Our study aims to identify differences in comorbidities and baseline characteristics between TTR cardiac amyloidosis patients with and without ICDs, and to compare survival outcomes between these two groups and other causes of non-ischemic cardiomyopathy (NICM), and to identify mortality predictors for TTR cardiac amyloidosis patients.

## 2. Methods

### 2.1 Study Design

This retrospective cohort study included patients with confirmed TTR cardiac amyloidosis, with and without ICDs, from 2001 to 2024 across all three primary Mayo Clinic sites (Arizona, Florida, and Minnesota campuses). Diagnosis of TTR-cardiac amyloidosis was based on either endomyocardial biopsy demonstrating amyloid deposition—prior to 2009 confirmed by immunohistochemistry with a negative hematologic workup, and after 2009 confirmed by mass spectrometry—or on positive technetium-99m PYP scintigraphy (Perugini Grade 2 or 3) obtained after 2016, in the absence of a monoclonal protein, as evidenced by negative serum and urine immunofixation and a normal serum free light-chain kappa/lambda ratio. Non-biopsy-proven diagnoses also required echocardiographic or CMR findings supportive of amyloid deposition [8]. NICM was identified by was identified based on the absence of significant coronary artery disease, as well as echocardiographic or cardiac MRI findings consistent with myocardial dysfunction. Subjects were identified using ICD-10 codes through an electronic data extraction system, followed by a comprehensive chart review to confirm the diagnosis. Patients with left ventricular assist devices were excluded. The study was approved by the Mayo Clinic Institutional Review Board (Approval No. 16-006578) and conducted in accordance with the ethical principles outlined in the Declaration of Helsinki. Given the retrospective nature of the study and the use of de-identified patient data, the requirement for informed consent was waived by the IRB. Data supporting the findings of this study are available from the corresponding author upon reasonable request.

### 2.2 Data Collection

In-depth chart reviews were conducted to collect baseline patient characteristics, including age at diagnosis, gender, race, and smoking status (categorized as active, former, or never smoker). Prior comorbidities such as diabetes mellitus (DM), hypertension (HTN), stroke or transient ischemic attack, deep vein thrombosis (DVT) or pulmonary embolism (PE), cancer, heart transplant, prior history of coronary artery disease (CAD), chronic kidney disease (CKD), and atrial fibrillation were recorded. Information on ICDs included the implantation date, indication, number of shocks (appropriate vs. inappropriate), reason for inappropriate shocks, and device-related complications. The episodes of ventricular arrhythmias requiring ICD shocks were individually reviewed and adjudicated as monomorphic VT or polymorphic VT/ventricular fibrillation with assessment of their successful termination or not. Of the 24 documented ICD shock episodes, full device data were available for 18 (75%). We performed a sensitivity analysis assuming that all six episodes with missing data represented failed terminations. Echocardiographic measurements included left ventricular ejection fraction, while laboratory values, such as N-terminal pro-B-type natriuretic peptide (NT-proBNP) and troponin T, were taken from the closest measurement to the date of TTR cardiac amyloidosis diagnosis. Due to the retrospective nature of the study and variability in clinical testing some biomarker data was not available for all patients. We performed sensitivity analysis and the findings were not sensitive to the method of handling missing data. The primary aim was to assess total mortality, while the secondary aims were evaluation of success rate of ICD shocks and identification of the predictors of mortality.

### 2.3 Statistical Analysis

Baseline characteristics, comorbidities, echocardiographic findings, and laboratory data were compared between patients with and without an ICD using *t*-tests or non-parametric tests for continuous variables, depending on data distribution, and Chi-square (χ^2^) tests for categorical variables. Cox proportional hazards regression was performed to identify predictors of mortality in the TTR cardiac amyloidosis population. Both univariable and multivariable analyses were conducted, with results reported as hazard ratios (HR) and corresponding 95% confidence intervals (CI). The multivariable analysis was adjusted for potential risk factors within the TTR cardiac amyloidosis population, including age, sex, history of CAD, history of stroke, CKD, cancer, troponin T levels >50 ng/L, NT-proBNP levels >3000 pg/mL, and LVEF <40%.

Survival probabilities were compared between cardiac amyloidosis patients with and without ICDs, and against a matched cohort of NICM patients with ICDs, using Kaplan–Meier survival curves and the log-rank test. To reduce confounding, propensity score matching was performed using a 1:4 nearest-neighbor algorithm without replacement and a caliper width of 0.1 times the standard deviation of the logit of the propensity scores. Matching was based on age, sex, hypertension, diabetes mellitus, prior stroke or TIA, DVT or PE, CKD, atrial fibrillation, cancer, coronary artery disease, and left ventricular ejection fraction. For time-to-event analyses, the index date in the Kaplan–Meier survival curve was set at the time of initial diagnosis of cardiac amyloidosis and NICM for all patients. For the Kaplan–Meier analysis of tafamidis use, the index date was defined as the date of the first tafamidis order for treated patients, and the date of ICD implantation for untreated patients. Patients were censored at the first occurrence of death, last clinical encounter, or at a maximum follow-up of 10 years. Continuous variables were summarized as mean ± standard deviation (SD) or median (interquartile range [IQR]), while categorical variables were reported as counts and percentages. Two-tailed *p*-values ≤ 0.05 were considered statistically significant. All statistical analyses were performed using SPSS Statistics (version 28.0.0.0 (190), IBM, Chicago, IL, USA).

## 3. Results

We identified 463 subjects diagnosed with biopsy-proven or PYP-confirmed TTR cardiac amyloidosis. The median age of the cohort was 74.5 years (IQR: 68.0, 80.0), with 92.9% of the patients being male. Table 1 summarizes the baseline characteristics, laboratory findings, and mortality of the study population. Among the total cohort, 257 patients (55.5%) did not receive an ICD), while 206 patients (44.5%) underwent ICD implantation.

**Table 1.** **The baseline characteristics, laboratory findings, and outcomes of the TTR cardiac amyloidosis population**.

Characteristics		Total	No-ICD implanted	ICD implanted	*p* value
		(N = 463)	(N = 257)	(N = 206)	
Age (years)		74.5 (68.0, 80.0)	77.0 (70.0, 81.0)	71.0 (66.0, 77.0)	0.001
Gender					0.181
	Male	430 (92.9%)	235 (91.4%)	195 (94.7%)	
	Female	33 (7.1%)	22 (8.6%)	11 (5.3%)	
Race					0.160
	White	408 (88.1%)	233 (90.7%)	175 (85.0%)	
	Black/African American	41 (8.9%)	16 (6.2%)	25 (12.1%)	
	Other	8 (1.7%)	5 (1.9%)	3 (1.5%)	
Smoking					0.540
	Never	258 (56.2%)	148 (58.0%)	110 (53.9%)	
	Current	6 (1.3%)	4 (1.6%)	2 (1.0%)	
	Former	195 (42.5%)	103 (40.4%)	92 (45.1%)	
CAD		95 (20.7%)	49 (19.4%)	46 (22.3%)	0.499
DM		117 (25.5%)	55 (21.8%)	62 (30.1%)	0.043
HTN		244 (53.3%)	115 (45.6%)	129 (62.6%)	0.001
Stroke or TIA		51 (11.1%)	29 (11.5%)	22 (10.7%)	0.779
CKD		241 (52.6%)	112 (44.4%)	129 (62.6%)	0.001
Atrial fibrillation		315 (68.8%)	169 (67.1%)	146 (70.9%)	0.476
Cancer		125 (27.3%)	68 (27.0%)	57 (27.7%)	0.658
Left ventricular ejection fraction (%)		51 (39, 60)	55 (47, 62)	43 (34, 55)	0.001
NT-proBNP (pg/mL)		1784 (818.5, 3832.5)	1503 (675.5, 3573.5)	2259 (1059.5, 4823.3)	0.007
Troponin T (ng/L)		47 (28, 73)	44 (29, 67)	54 (25, 80)	0.102
Cardiac transplant		47 (10.2%)	6 (2.3%)	41 (19.9%)	0.001

Continuous variables are reported as median (inter-quartile range), and categorical variables as n (%). Abbreviations: CAD, coronary artery disease; CKD, chronic kidney disease; DM, diabetes mellitus; HTN, hypertension; ICD, implantable cardioverter-defibrillator; TIA, transient ischemic attack; NT-proBNP, N-terminal pro-B-type natriuretic peptide.

### 3.1 Baseline Characteristics in TTR Cardiac Amyloidosis Patients

The ICD-implanted group was significantly younger than the non-ICD group, with a median age of 71.0 years compared to 77.0 years (*p* = 0.001). The study population was predominantly male (92.9%), and the gender distribution did not significantly differ between groups (*p* = 0.181). In terms of racial distribution, 88.1% of the cohort were White, 8.9% were Black/African American, and 1.7% identified as Other. Although the ICD group had a slightly higher proportion of Black/African American patients (12.1% vs. 6.2%), this difference was not statistically significant (*p* = 0.160).

Regarding cardiovascular risk factors, patients in the ICD group had a significantly higher prevalence of HTN (62.6% vs. 45.6%, *p* = 0.001) and CKD (62.6% vs. 44.4%, *p* = 0.001). The prevalence of DM was also higher in the ICD group (30.1% vs. 21.8%, *p* = 0.043). Conversely, the prevalence of CAD (22.3% vs. 19.4%, *p* = 0.499), stroke/transient ischemic attack (10.7% vs. 11.5%, *p* = 0.779), and prevalence of atrial fibrillation (70.9% vs. 67.1%, *p* = 0.476) did not significantly differ between groups.

In terms of cardiac function, the median left ventricular ejection fraction was lower in the ICD groups (43% vs. 54%, *p* = 0.007), while NT-proBNP levels were significantly higher in ICD recipients (2259.0 pg/mL vs. 1503.0 pg/mL, *p* = 0.007), suggesting a greater burden of heart failure in this group.

### 3.2 Patients With ICDs

Among 206 patients who received an ICD, the majority (157 patients, 76.2%) underwent implantation for primary prevention of sudden cardiac death, while 48 patients (23.3%) received an ICD for secondary prevention.

As shown in Table 2, shock therapy was delivered to 13.6% of ICD recipients, with a slightly higher rate in the secondary prevention (14.6%) compared to the primary prevention group (12.7%). Appropriate shock therapy occurred in 10.6% of cases, with monomorphic ventricular tachycardia (VT) being the most common arrhythmia requiring intervention. Among patients receiving appropriate shocks, 8.6% had VT, while 2% had ventricular fibrillation. In patients who received appropriate shock therapy, 75% achieved successful dangerous arrhythmia termination, assuming all missing cases were unsuccessful. The median VT rate was 200 beats per minute (range 162–239 bpm). Lastly, all documented causes of deaths were not attributed to arrhythmia.

**Table 2.** **Outcomes of implantable cardioverter-defibrillators in cardiac amyloidosis**.

Outcomes			Implanted ICD	Primary prevention due to HF	Secondary prevention
			(N = 206)	(N = 157)	(N = 48)
Shock Therapy			28 (13.6%)	20 (12.7%)	7 (14.6%)
	Appropriate shock		22 (10.7%)	15 (9.5%)	6 (12.5%)
		VF	4 (2%)	4 (2.5%)	0
		VT	18 (8.7%)	11 (7%)	6 (12.5%)
	VT rate (bpm)		200 (161.5, 238.5)		
	Success therapy		18/24 (75%)		
	VT storm		3 (1.5%)		
	Inappropriate shock		6 (3%)	5 (3.2%)	1 (2.1%)
		Supraventricular source	5 (2.5%)	4 (2.5%)	1 (2.1%)
		Oversensing/under-sensing	1 (0.5%)	1 (0.6%)	0
		Device malfunction	0		
ICD complications			12 (5.8%)		
		Lead-related complications	10 (4.8%)		
		Infection	2 (1%)		
Mortality secondary to EMD			1 (0.4%)		

Continuous variables are reported as median (inter-quartile range), and categorical variables as n (%). Abbreviations: EMD, electromechanical dissociation; HF, heart failure; VF, ventricular fibrillation; VT, ventricular tachycardia.

A small proportion of patients (1.5%) experienced VT storm, requiring multiple ICD interventions within 24 hours. Inappropriate shocks occurred in 3% of all ICD recipients, with 16% of those cases involving patients with a single-chamber device. The primary cause of inappropriate shocks in these patients was supraventricular arrhythmias (2.5%) with a smaller proportion caused by oversensing or undersensing (0.5%). No cases of device malfunction were reported.

ICD complications occurred in 5.8% of cases, with lead-related complications that includes lead erosion, impingement, and malfunction making up 4.8% and general infections accounting for 1%.

### 3.3 Survival Analysis and Mortality Predictors

**Fig. 1.**
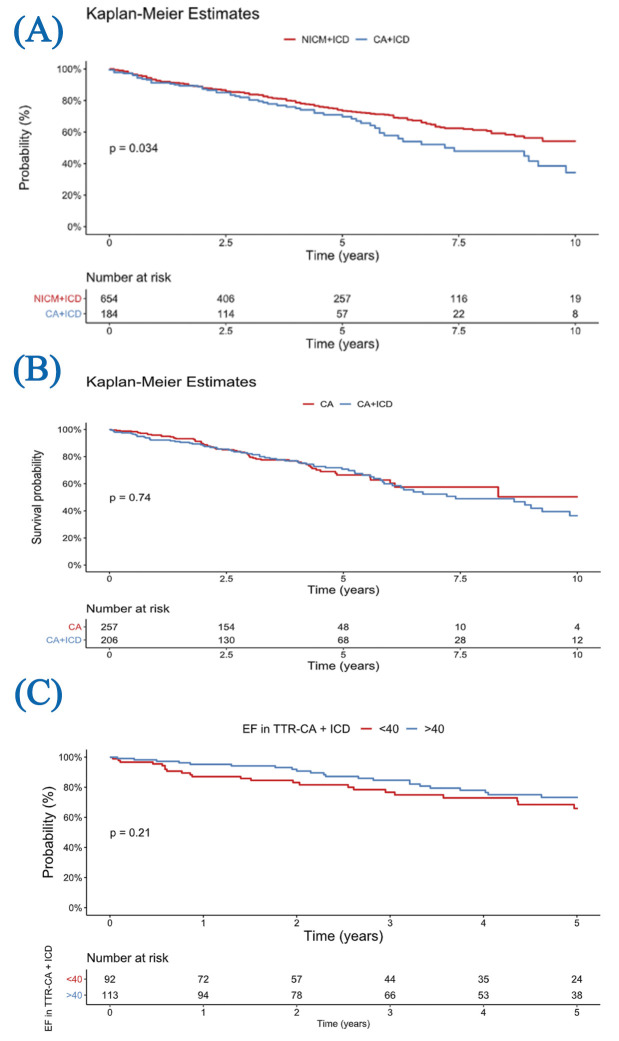
**Kaplan-Meier survival curves**. (A) Survival from diagnosis to death in cardiac amyloidosis and NICM patients with ICDs. (B) Survival from diagnosis in patients with cardiac amyloidosis, stratified by ICD implantation. (C) Subgroup analysis of ICD-treated TTR cardiac amyloidosis patients stratified by EF >40% vs <40%. NICM, non-ischemic cardiomyopathy; CA, cardiac amyloidosis; TTR, transthyretin; EF, ejection fraction.

We compared survival outcomes between patients with TTR cardiac amyloidosis and a matched NICM cohort who had ICDs (Fig. 1A). Table 3 illustrates the variables used for matching. The median follow up of the matched NICM cohort was 7.1 years (IQR: 4.6–8.8 years) and 6.8 years (IQR: 4.5–9.0 years) for the TTR cardiac amyloid group. Over a 10-year follow-up period, survival differed significantly (*p* = 0.034). NICM patients with ICDs exhibited significantly better survival than TTR cardiac amyloidosis patients.

**Table 3.** **Baseline Characteristics of the overall and matched cohort with a comparison between patients receiving ICD for Cardiac amyloidosis and nonischemic cardiomyopathy**.

Characteristics	Before 1:4 propensity score matching			After 1:4 propensity score matching		
	CA + ICD	NICM + ICD	SMD	CA + ICD	NICM + ICD	SMD
	(N = 206)	(N = 5462)		(N = 184)	(N = 654)	
Age (at diagnosis), y	71.5 (66, 77)	64 (54, 73)	0.876	71 (65, 77)	72 (64, 79)	–0.089
Male sex	195 (94.7%)	3797 (69.2%)	1.334	176 (95.7%)	627 (95.9%)	–0.027
HTN	129 (62.6%)	3851 (70.2%)	–0.157	117 (63.6%)	426 (65.1%)	–0.025
DM	62 (30.1%)	2401 (43.8%)	–0.301	57 (31.0%)	216 (33.0%)	–0.022
Stroke/TIA	22 (10.7%)	878 (16.0%)	–0.144	21 (11.4%)	71 (10.9%)	0.012
DVT/PE	15 (5.8%)	682 (12.4%)	–0.175	15 (8.2%)	55 (8.4%)	0.000
CKD	129 (62.6%)	3414 (62.2%)	0.039	117 (63.6%)	427 (65.3%)	–0.060
Atrial fibrillation	146 (70.9%)	3698 (67.4%)	0.100	131 (71.2%)	489 (74.8%)	–0.074
Cancer	57 (27.7%)	1792 (32.7%)	–0.080	54 (29.3%)	196 (30.0%)	0.005
CAD	46 (22.3%)	1848 (33.7%)	–0.261	43 (23.4%)	161 (24.6%)	–0.013
EF (%)	43 (34, 55)	26 (17, 37)	1.102	42 (34, 52)	41 (30, 50)	0.045

Abbreviations: SMD, standardized mean difference; DVT, deep vein thrombosis; PE, pulmonary embolism. *Continuous variables were reported as median (IQR) or mean ± SD, categorical variables were reported as N (%).

When analyzing only patients with TTR cardiac amyloidosis, follow-up duration was 7.4 years (IQR: 5.3–9.2 years) for the non-ICD group and 6.8 years (IQR: 4.5–9.0 years) for the ICD group. There was no significant difference in survival between those who received an ICD and those who did not (*p* = 0.74; Fig. 1B). The survival curves for both TTR cardiac amyloidosis patient groups closely paralleled each other, showing the minimal impact of ICD therapy in this group. In a subgroup analysis of patients with TTR cardiac amyloidosis who received an ICD, five-year survival was not significantly different between those with EF >40% and those with EF <40% (*p* = 0.21; Fig. 1C).

**Fig. 2.**
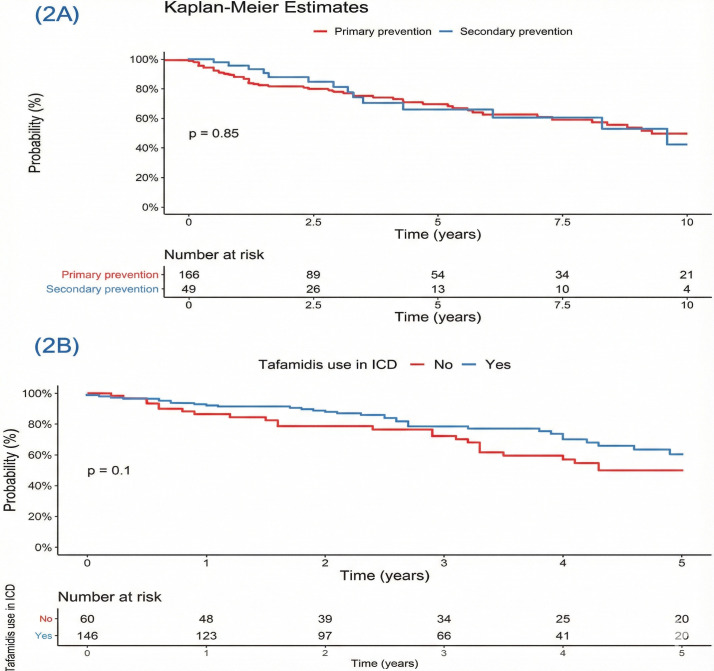
**Kaplan-Meier survival curves**. (A) Survival from diagnosis to death in cardiac amyloidosis based on indication for ICD. (B) Survival from diagnosis in patients with cardiac amyloidosis, stratified by tafamidis use in patients with an ICD.

A TTR-cardiac amyloid subgroup survival analysis was done. Fig. 2A illustrates the survival comparison between patients with transthyretin cardiac amyloidosis (CA-TTR) who received an ICD for primary prevention versus those who received it for secondary prevention; no significant difference in survival was observed between the groups (*p* = 0.85). Fig. 2B compares survival in patients treated with tafamidis versus those not on the medication, which also did not reach statistical significance (*p* = 0.10).

In the TTR cardiac amyloidosis group, we identified 23 patients with the V122I variant, who had a median survival time of 1.87 years (IQR: 0.43–3.48). In contrast, among 440 patients without the V122I variant, the median survival was 3.24 years (IQR: 1.74–5.02). The statistically significant difference (*p* = 0.007) suggests that the V122I variant is associated with a shorter survival time within the TTR cardiac amyloidosis population.

### 3.4 Mortality Predictors

Table 4 presents the Cox regression analysis identifying key predictors of mortality in TTR cardiac amyloidosis. Significant risk factors included older age (HR: 1.048 per year, *p* = 0.001), CKD (HR: 1.637, *p* = 0.029), elevated troponin T >50 ng/L (HR: 1.594, *p* = 0.031), and NT-proBNP >3000 pg/mL (HR: 1.514, *p* = 0.05). The strongest predictor was reduced left ventricular ejection fraction (<40%), nearly doubling mortality risk (HR: 1.935, *p* = 0.003). Other factors analyzed in Table 4, including sex, CAD, stroke and TIA, cancer, and ICD implantation did not significantly affect mortality (*p* > 0.05).

**Table 4.** **Predictors of mortality in patients with TTR cardiac amyloidosis (Cox regression analysis)**.

Variable	Hazard Ratio (95% CI)	*p* value
Age	1.048 (1.021–1.076)	0.001
Sex	0.552 (0.235–1.297)	0.173
History of CAD	1.051 (0.654–1.687)	0.837
Stroke or TIA	1.174 (0.662–2.085)	0.583
CKD	1.637 (1.052–2.550)	0.029
Cancer	1.178 (0.772–1.797)	0.448
Troponin >50 ng/L	1.594 (1.046–2.429)	0.031
Pro-BNP >3000 pg/mL	1.514 (1.091–2.304)	0.050
Ejection Fraction <40%	1.935 (1.251–2.994)	0.003
ICD Implantation	0.932 (0.591–1.470)	0.763

## 4. Discussion 

This study represents one of the largest cohorts of patients with TTR cardiac amyloidosis, with or without an ICD. Patients with TTR cardiac amyloidosis who received an ICD did not demonstrate a survival benefit compared to those without an ICD and had significantly worse survival than a matched cohort of patients with NICM and ICDs. Subgroup analysis within the TTR cardiac amyloidosis population revealed no difference in mortality between patients who received ICDs for primary versus secondary prevention, nor between those treated with tafamidis versus not. Notably, all documented deaths in the TTR cardiac amyloidosis cohort were non-arrhythmic in nature. Our study highlights that CKD, elevated NT-proBNP, and advanced age point to pump failure as the dominant mode of death. Independent predictors of mortality included older age, reduced left ventricular ejection fraction, elevated troponin, and NT-proBNP levels.

### 4.1 Baseline Characteristics in TTR Cardiac Amyloidosis Patients

When comparing TTR cardiac amyloidosis patients with ICD to those without ICD, the ICD group had a higher prevalence of CKD, HTN, and T2DM. CKD was associated with a higher prevalence of heart failure (HF), which was a leading cause of hospitalization and mortality in these patients [9]. CKD patients were also at greater risk for ventricular fibrillation, VT, and sudden cardiac death, which accounts for 25–29% of all-cause mortality in hemodialysis patients [10]. Additionally, HTN increases the risk of HF and ventricular arrhythmias. HTN also contributes to left ventricular thickening and remodeling, which can predispose to these complications [11]. Similarly, DM type 2 and obesity both raise the risk of HF, with sudden cardiac death being 3–8 times more common in diabetes patients [12, 13]. In our study, NT-proBNP levels were higher in the TTR cardiac amyloidosis group with ICD, as elevated NT-proBNP is often associated with worse clinical status and decompensated HF [14]. These findings may explain the observed differences when comparing TTR cardiac amyloidosis patients with and without ICDs. The significant variables increase the risk of HF and ventricular arrhythmias, leading to a higher likelihood of ICD implantation for primary and secondary prevention. Additionally, this may be explained by the more extensive disease in ICD patients due to multi-organ involvement and failure [15].

### 4.2 Shocks and Mortality Outcomes

Shock therapy was delivered to 28 (13.6%) of ICD recipients. Importantly, appropriate shock therapy occurred in 22 (10.6%) of cases, with monomorphic VT being the most common arrhythmia requiring intervention. The ICD complication rate in our cohort was 5.8%, which aligns with prior reports in cardiac amyloidosis patients, including rates of 5.7%–7.2% in recent studies [16, 17]. This should be put into perspective when evaluating the overall utility of ICD therapy in this population. Notably among the patients with available data, all patients who received VT-directed shock therapy achieved successful termination, demonstrating that ICD shocks are effective in this patient population. This contrasts with a prior study suggesting that ICDs may not significantly improve outcomes in cardiac amyloidosis due to the high rate of electromechanical dissociation [18]. However, a prior study reported a high success rate of 80% for ICD shocks in these patients [17]. Despite this high success rate for ICD shocks, survival outcomes for TTR cardiac amyloidosis patients, regardless of ICD presence, were worse compared to other NICM, suggesting that death in these cardiac amyloidosis patients may be primarily due to non-arrhythmic causes. Even within the TTR cardiac amyloidosis group there was no difference in the survival outcome from ICD, and that finding aligns with previous studies that failed to show clear mortality benefit by the presence of an ICD [19]. Additionally, our cohort demonstrated no difference in survival among TTR cardiac amyloidosis patients based on ICD indication or tafamidis use. These findings reinforce that ICD therapy offers limited survival benefit in this population, as even a history of ventricular arrhythmia did not improve mortality outcomes. While tafamidis is well-established as a disease-modifying therapy that slows progression and improves survival in TTR cardiac amyloidosis, its use did not translate into improved survival among patients already selected for ICD therapy [20]. This suggests that both device therapy and pharmacologic treatment may have limited impact once advanced cardiac dysfunction is established.

### 4.3 Mortality Predictors

Several factors have been identified in our study as predictors of mortality in TTR cardiac amyloidosis patients, including older age, CKD, elevated troponin T (>50 ng/L), NT-proBNP >3000 pg/mL, and a reduced ejection fraction (<40%). These findings are consistent with previous studies. One study demonstrated that older age (above 80 years), elevated NT-proBNP (greater than 4200 pg/mL), and elevated cardiac troponin (greater than 92 ng/L) were independent prognostic factors [21]. The combination of these variables identified standard- and high-risk patients (all above the cutoff levels) with median survival of 57 and 17 months, respectively [17]. Another study showed that older age, higher NYHA functional class, and elevated BNP levels were significant mortality predictors [22].

Our study highlighted differences in baseline characteristics between TTR cardiac amyloidosis patients with ICDs versus those without ICDs. Many implants occur too late in the disease course, so older patients with ICDs were more likely to have comorbidities like CKD and higher Pro-BNP levels at presentation, suggesting a worse prognosis. Although these patients experienced shocks, primarily for ventricular tachycardia, with a (75%–100%) success rate based on available data, mortality did not decrease in this group, highlighting ICD therapy does not improve pump function. The reduced left ventricular ejection fraction, which was the strongest predictor of mortality, nearly doubled the risk for death in these patients. Put together with prior studies, the findings suggest that the mode of death is related to disease progression resulting in pump failure [19, 23].

## 5. Limitations

This study has several limitations. First, its retrospective design across three sites of a quaternary referral institution may limit generalizability. Heterogeneity is inherent in a retrospective design. As an observational study, it is subject to selection bias, and randomized controlled trials are needed for more robust comparisons between transthyretin cardiac amyloidosis (TTR-CA) patients with and without ICDs. Use of ICD-10 codes may have led to under- or over-reporting, though this was mitigated by thorough database review. Temporal bias remains an issue and it is unclear if amyloid patients from the earlier part of the cohort had more severe disease at the time of diagnosis.

ICD technology evolved over the two-decade study period, including waveform and algorithm improvements, which may have affected shock efficacy. Tafamidis exposure was inconsistent due to its recent approval, and inverse-probability weighting was not applied to address selection bias for ICD implantation. Although all documented deaths were not attributed to arrhythmia, most patients did not have a documented cause of death. Patients with missing biomarker data were included in analyses. Nuclear imaging techniques such as technetium-99m PYP scans were not routinely performed in earlier years. In such cases, diagnosis was based on available contemporary standards, including clinical criteria, echocardiographic features, cardiac magnetic resonance imaging (MRI) findings, biopsy results, and genetic testing when applicable. Temporal trends in diagnostic approaches were acknowledged as a study limitation and were reviewed during data abstraction to ensure consistent application of inclusion criteria across the time frame. Limited access to genetic testing and unstratified CKD severity may also have influenced the findings.

## 6. Conclusions 

In conclusion, our study highlights the lack of survival benefit of ICD implantation in patients with TTR cardiac amyloidosis despite a high rate of successful termination of ventricular arrhythmia. Key predictors of mortality include reduced ejection fraction and elevated biomarkers. These findings suggest that current ICD implantation practices may not confer an overall survival advantage, particularly in older patients with impaired pump function, where the overall benefit may be limited. Further prospective studies are needed to better understand the role of ICD in TTR cardiac amyloidosis patients and to improve treatment strategies for this patient population.

## Availability of Data and Materials

The data underlying this article will be shared on reasonable request to the corresponding author.
